# Urinary peptidomics reveals proteases involved in idiopathic membranous nephropathy

**DOI:** 10.1186/s12864-021-08155-3

**Published:** 2021-11-24

**Authors:** Baoxu Lin, Jianhua Liu, Yue Zhang, Yabin Wu, Shixiao Chen, Yibo Bai, Qiuying Liu, Xiaosong Qin

**Affiliations:** Department of Laboratory Medicine, Shengjing Hospital of China Medical University, No. 36 Sanhao Street, Heping District, 110004 Shenyang, P. R. China

**Keywords:** Urinary peptidomics, Idiopathic membranous nephropathy, Proteases

## Abstract

**Background:**

Idiopathic membranous nephropathy (IMN) is a cause of nephrotic syndrome that is increasing in incidence but has unclear pathogenesis. Urinary peptidomics is a promising technology for elucidating molecular mechanisms underlying diseases. Dysregulation of the proteolytic system is implicated in various diseases. Here, we aimed to conduct urinary peptidomics to identify IMN-related proteases.

**Results:**

Peptide fingerprints indicated differences in naturally produced urinary peptide components among 20 healthy individuals, 22 patients with IMN, and 15 patients with other kidney diseases. In total, 1,080 peptide-matched proteins were identified, 279 proteins differentially expressed in the urine of IMN patients were screened, and 32 proteases were predicted; 55 of the matched proteins were also differentially expressed in the kidney tissues of IMN patients, and these were mainly involved in the regulation of proteasome-, lysosome-, and actin cytoskeleton-related signaling pathways. The 32 predicted proteases showed abnormal expression in the glomeruli of IMN patients based on Gene Expression Omnibus databases. Western blot revealed abnormal expression of calpain, matrix metalloproteinase 14, and cathepsin S in kidney tissues of patients with IMN.

**Conclusions:**

This work shown the calpain/matrix metalloproteinase/cathepsin axis might be dysregulated in IMN. Our study is the first to systematically explore the role of proteases in IMN by urinary peptidomics, which are expected to facilitate discovery of better biomarkers for IMN.

**Supplementary Information:**

The online version contains supplementary material available at 10.1186/s12864-021-08155-3.

## Background

Idiopathic membranous nephropathy (IMN), a known cause of nephrotic syndrome, is a public health concern estimated to affect 1–18 people in a population of 100,000 globally [1]. Proteinuria has been reported in 75–85 % of patients with IMN, and 40–60 % of IMN patients may progress to end-stage renal failure within 5–20 years [1]. Thus, urinary protein level is an important indication for diagnosing, planning the treatment, and evaluating the progress of IMN [2].

Proteinuria and related symptoms are considered clinical manifestations of podocyte injury in IMN [3]. The involvement of kidney injury in IMN was identified by pathological examination of kidney tissue using immunofluorescence and electron microscopy in the 1940 s [4]. However, the molecular mechanism underlying kidney injury remains unclear. In 2009, an anti-phospholipase A2 receptor (anti-PLA2R) antibody was discovered in the sera of IMN patients [1]. Since then, it has been used in the diagnosis and therapeutic targeting of IMN, with about 70–80 % of patients being positive for this marker [5]. Nevertheless, the mechanism underlying anti-PLA2R antibody production in these patients has not been fully clarified. Therefore, in this context, the main goal is to unravel the pathogenic molecular mechanism underlying kidney damage.

A complex protease network is at play in podocytes [6–8], and protease-dependent signaling pathways are reportedly related to inflammation and tissue remodeling in kidney diseases [9]. Therefore, proteases are becoming increasingly important in targeted therapy of renal diseases [3].

Large-scale research on proteins is termed proteomics, whereas that on naturally occurring peptides is termed peptidomics [9, 10]. High-resolution mass spectrometry (MS), especially data-independent acquisition MS (DIA-MS), has enabled broad application of proteomics in medical science. Urinary peptidomics is a promising technology to elucidate the pathogenesis of IMN [11], because the urine may contain low-molecular-weight peptides produced by endogenous protease activity in the kidneys [12, 13], and the action mechanism of proteases in kidney diseases, which has been mainly reported in diabetic nephropathy [14, 15].

A typical example of application of urinary peptidomics is development of urinary peptide biomarkers, such as the CKD 273 classifier that has been proposed for diagnosis of chronic kidney disease and is undergoing clinical trials [16]. Recent studies explored the mechanisms underlying urinary peptide production in gastric cancer [17] and Parkinson’s disease [18], further indicating that urine reflects changes in biological pathways at the tissue level.

In this study, we aimed to elucidate the biological processes and signaling pathways involved in IMN-related protein dysregulation and tissue injury by analyzing naturally produced urinary peptides differentially expressed in IMN patients, identifying proteins matched to these peptides, and predicting and validating the proteases involved in the production of these peptides. Our findings are expected to serve as a reference for studies on the pathological mechanism underlying IMN and discovery of related biomarkers.

## Results

### Clinical characteristics of subjects

Clinical information of subjects is summarized in Table 1. The estimated glomerular filtration rate (eGFR) was calculated with CKD-EPI (Chronic Kidney Disease Epidemiology Collaboration) Cystatin C formula (2012). eGFR of IMN and other nephropathy groups was lower than that of the HC group.

**Table 1:** Comparison of demography and eGFR between IMN, OD, and HC groups

	HC	IMN	OD	*P*
Sex (Male/Female)^a^	10/10	13/9	8/7	> 0.05
Age (mean ± SD)^b^	47.2 ± 11.9	50.2 ± 10.7	42.0 ± 15.0	0.84
eGFR/mL/min/1.73 m^2^ (mean ± SD)^b*^	113.8 ± 19.4	79.8 ± 22.1	53.7 ± 25.9	< 0.01

^*^ Indicates there is significant difference among HC, IMN and OD groups, *P* < 0.05;

^a^ and ^b^ indicate that Chi-Square test and one-way analysis of variance were applied, respectively

eGFR, estimated glomerular filtration rate; IMN, idiopathic membranous nephropathy; OD, other kidney disease; HC, healthy control; SD, standard deviation

### Global characteristics of urinary peptidomics of IMN

**Fig. 1:**
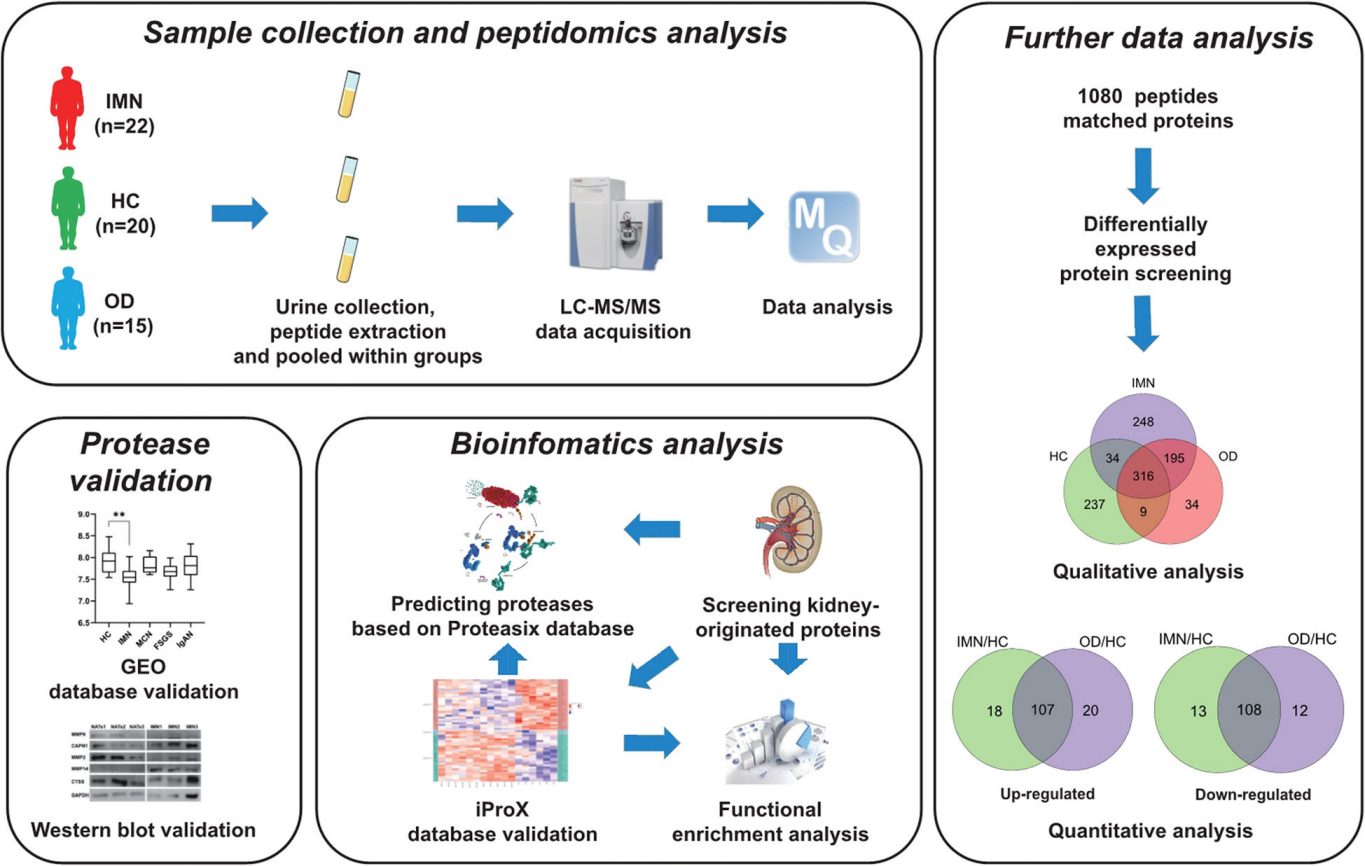
Workflow of the study. First, urine samples were collected from subjects; next, peptides were extracted from the samples and analyzed with liquid chromatography-tandem mass spectrometry (LC-MS/MS). Peptides differentially expressed in patients with idiopathic membranous nephropathy (IMN) and matched proteins were identified. Bioinformatics analyses were performed to predict kidney-originated proteins and reveal the expression pattern in kidneys based on the iProX database. Functional enrichment analysis was conducted using the Proteasix database to predict proteases. Finally, proteases were validated via Gene Expression Omnibus (GEO) database and western blot analyses. HC, healthy control; OD, other kidney disease

**Fig. 2:**
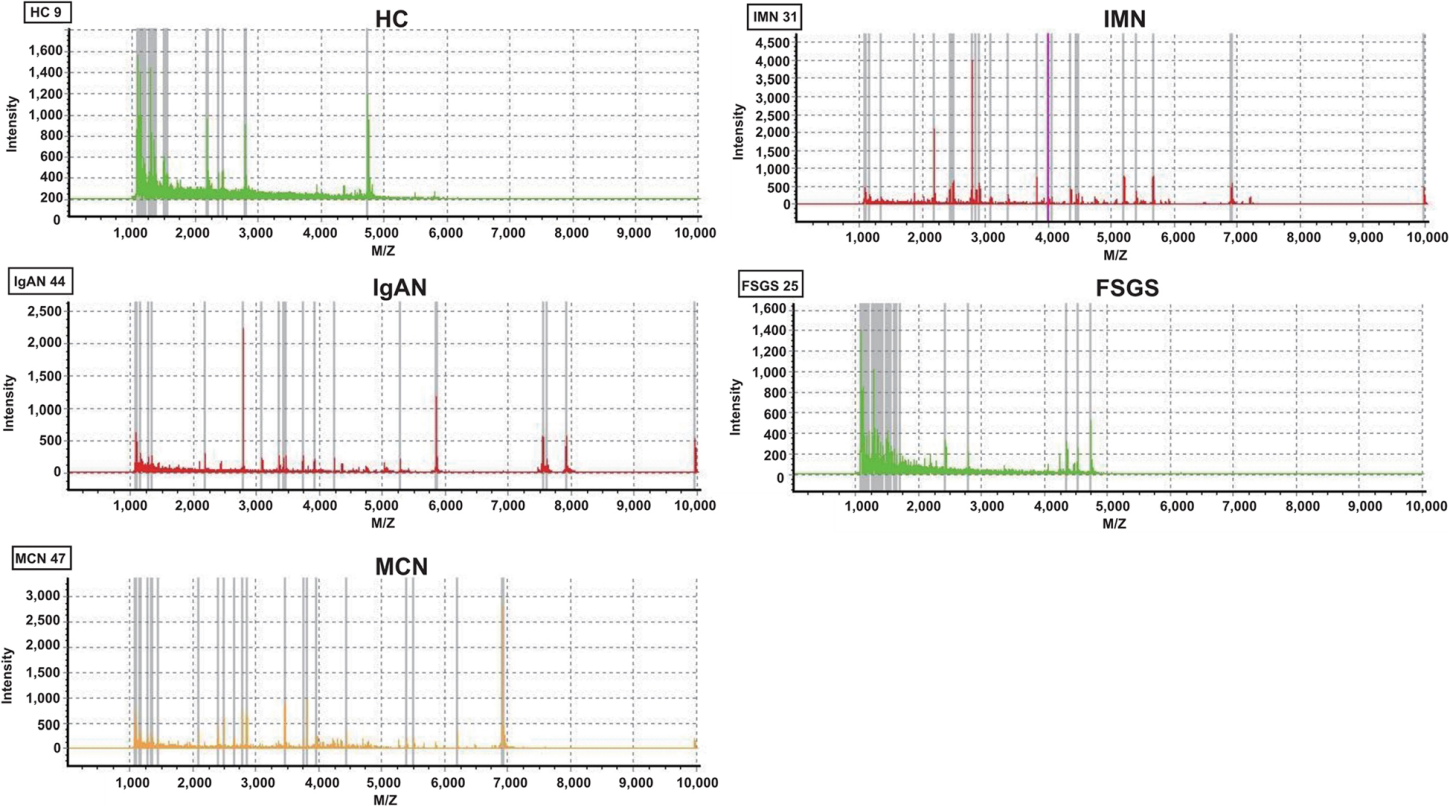
Urinary peptide fingerprints. The X-axis indicates detected peptides smaller than 10,000 kDa, and the Y-axis denotes the intensity of the peptide signals. The peptide components differed among healthy control, idiopathic membranous nephropathy (IMN), IgA nephropathy (IgAN), focal segmental glomerulosclerosis (FSGS), and minimal change disease (MCN) groups

The workflow of the study is shown in Fig. 1. Peptide fingerprint of each sample for all groups was profiled using MALDI-TOF MS; one sample representative of each group is shown in Fig. 2, which shows the differences in the urinary peptide fingerprints among healthy individuals and patients with kidney disease. Altogether, these results proved that we successfully extracted meaningful peptides from the urine samples.

In DIA-MS, 1080 peptide-matched proteins were identified, 559 proteins were quantified. In a qualitative analysis of differentially expressed proteins (DEPs) in IMN, 248 proteins were identified as unique to IMN patients. In quantitative analysis, 316 proteins were identified in HC, IMN, and OD groups. The comparison of the IMN and HC groups (IMN/HC) revealed 125 (fold change > 1.5) and 121 (fold change < 1.5) upregulated and downregulated proteins, respectively, whereas the comparison between the OD and HC groups (OD/HC), identified 127 (fold change > 1.5) and 120 (fold change < 1.5) upregulated and downregulated proteins, respectively. The shared upregulated and downregulated proteins in the two comparisons were 18 and 13, respectively. Thus, 279 IMN DEPs were selected (Fig. 1).

### Kidney-originated urinary proteins and GO enrichment

**Fig. 3:**
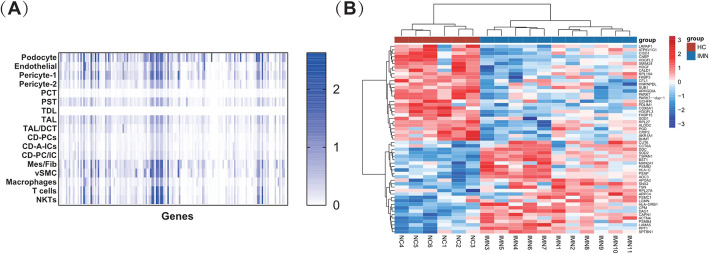
Heat maps showing (**A**) the expression pattern of genes encoding the 158 kidney-originated proteins in single renal cells and (**B**) 55 differentially expressed proteins (DEP) in kidney tissue from patients with idiopathic membranous nephropathy (IMN) based on iProX database analysis

Among 279 IMN DEPs, 158 kidney-originated urinary proteins were predicted based on sc-RNA seq database analysis (Fig. 3 A). Protein expression patterns indicated that most proteins might be from podocytes.

**Fig. 4:**
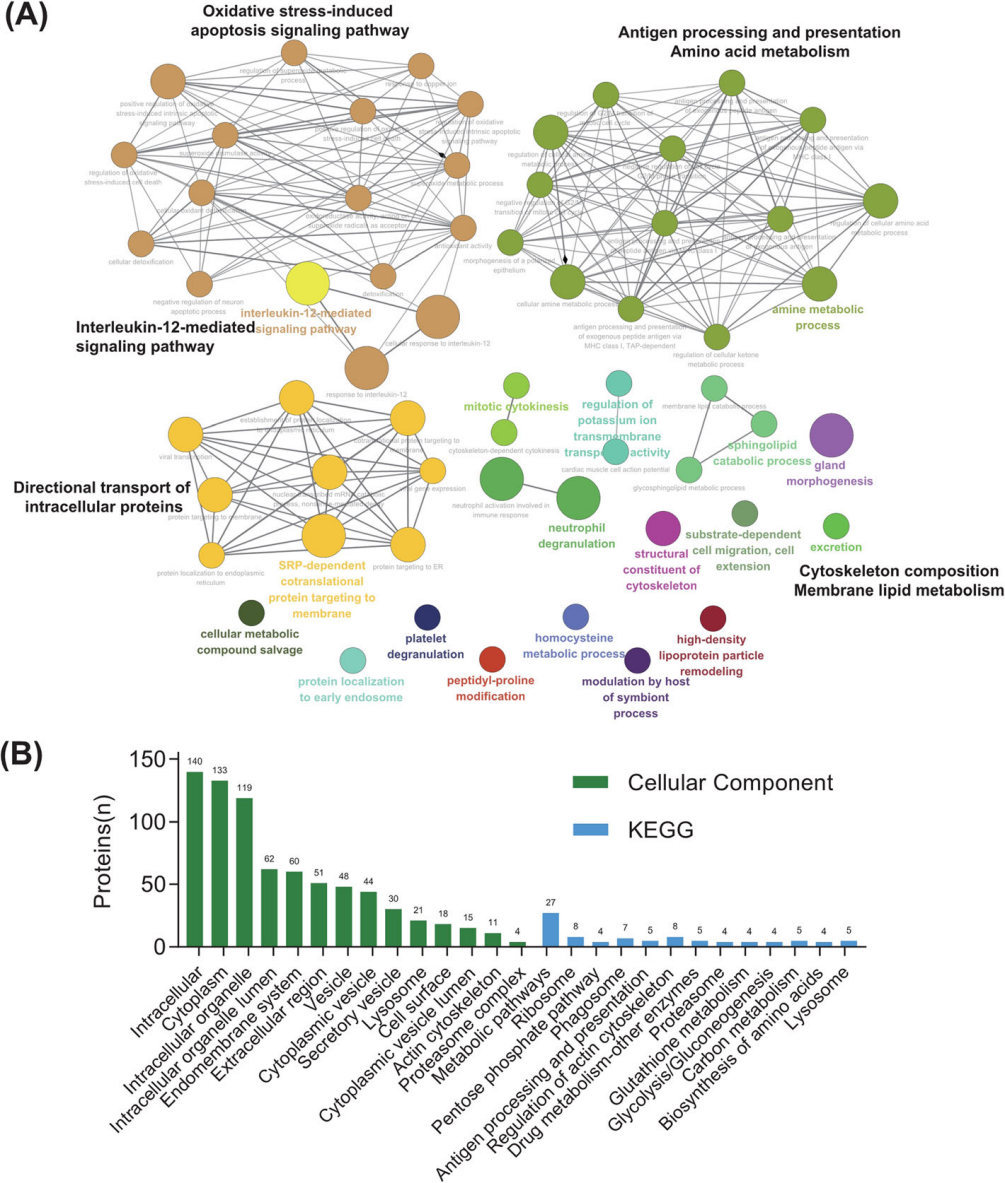
Gene Ontology (GO) enrichment analysis of kidney-derived urine proteins. **A** Interaction network of biological processes. **B** Cellular components and Kyoto Encyclopedia of Genes and Genomes (KEGG) signaling pathways

ClueGO software was used to analyze the interaction network of biological processes of 158 DEPs and revealed that these proteins were related to 60 biological processes, mainly divided into four categories (Fig. 4 A): (1) signaling pathways related to cellular pathological changes; (2) biological processes related to cell metabolism and cell activity; (3) biological processes related to intracellular protein transport; and (4) others. Among these, biological processes include oxidative stress and oxidative stress-induced apoptosis signaling pathway and interleukin-12-mediated signaling pathway. The second type comprises antigen processing and presentation, and amino acid metabolism. The third type contains the directional transport of intracellular proteins to the cell membrane and endoplasmic reticulum. The fourth type included cytoskeleton composition and membrane lipid metabolism.

The cellular localization of 158 DEPs and the involved signaling pathways were analyzed using DAVID. The results showed that the cellular localization of these proteins mainly included cell surface, cytoplasm, intracellular organelles, intracellular organelle lumen, intimal system, extracellular region, cell vesicle, lysosome, proteasome complex, etc. (Fig. 4B). The signaling pathways involved by these proteins included proteasome, lysosome, regulation of actin cytoskeleton, antigen processing and presentation, ribosome and metabolic pathway, etc. (Fig. 4B). These results indicate that the IMN DEPs may reflect the pathological changes in various metabolic processes.

### Validation of IMN DEPs in kidney tissue

To explore the expression of urinary peptide-matched proteins in the kidney, we investigated 158 urinary IMN DEPs using the iProX proteomics database. We obtained the quantitative analysis data of 131 urinary DEPs; 55 DEPs were identified between HC and IMN groups, of which 28 were upregulated and 27 were downregulated in IMN patients (Fig. 3B).

**Fig. 5:**
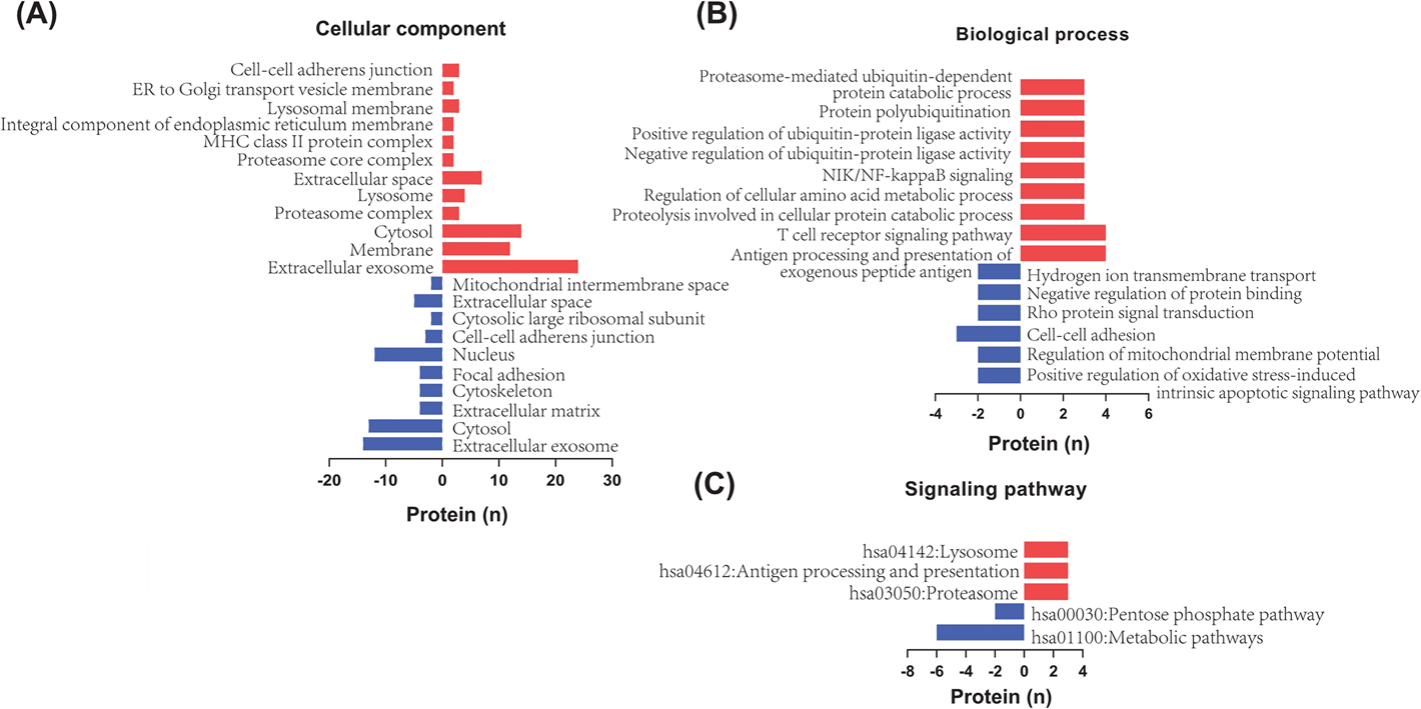
Functional annotation of differentially expressed proteins validated in iProX. **A** Cellular components; (**B**) Biological processes; (**C**) Signaling pathways. Upregulated and downregulated pathways are indicated by red (right) and blue (left) bars

GO enrichment analysis showed that both upregulated and downregulated proteins were related to protein degradation. The biological processes related to the upregulated proteins included proteolysis and proteasome-mediated protein catabolism. The biological processes related to the downregulated proteins included cell adhesion and protein binding (Fig. 5B). The cellular localization of upregulated proteins was in the proteasome and lysosome. Downregulated proteins were located in the extracellular matrix and cytoskeleton (Fig. 5 A). Upregulated proteins participated in lysosomal and proteasome signaling pathways, whereas downregulated proteins were involved in metabolic signaling pathways (Fig. 5 C). These results suggested that some damaging changes may occur in the kidneys of IMN patients, such as enhanced proteolysis and decreased intercellular adhesion. The peptides associated with these proteins may be released into the urine; thus, the level of peptides in the urine may reflect the severity of kidney tissue injury.

### Protease prediction and validation in the glomerulus

To link urinary peptides with proteases, Proteasix database analysis was used to predict proteases that may cleave the 158 proteins identified in this study. In total, 32 proteases were predicted (Table 2).

**Table 2:** List of 32 proteases predicted using the Proteasix tool

Protein symbol	Protein name	Protein symbol	Protein name
CAPN1	calpain 1	GZMB	granzyme B
CAPN2	calpain 2	GZMK	granzyme K
CAPN3	calpain 3	GZMM	granzyme M
CASP1	caspase 1	HPN	hepsin
CASP2	caspase 2	LGMN	legumain
CASP3	caspase 3	MEP1A	meprin A subunit alpha
CASP6	caspase 6	MEP1B	meprin A subunit beta
CASP9	caspase 9	MMP1	matrix metallopeptidase 1
CTSB	cathepsin B	MMP14	matrix metallopeptidase 14
CTSG	cathepsin G	MMP2	matrix metallopeptidase 2
CTSK	cathepsin K	MMP3	matrix metallopeptidase 3
CTSL	cathepsin L	MMP7	matrix metallopeptidase 7
CTSS	cathepsin S	ST14	suppression of tumorigenicity 14
DAG1	dystroglycan 1	TMPRSS11E	transmembrane protease, serine 11E
ELANE	elastase, neutrophil expressed	TMPRSS6	transmembrane protease, serine 6
GZMA	granzyme A	TMPRSS7	transmembrane protease, serine 7

**Fig. 6:**
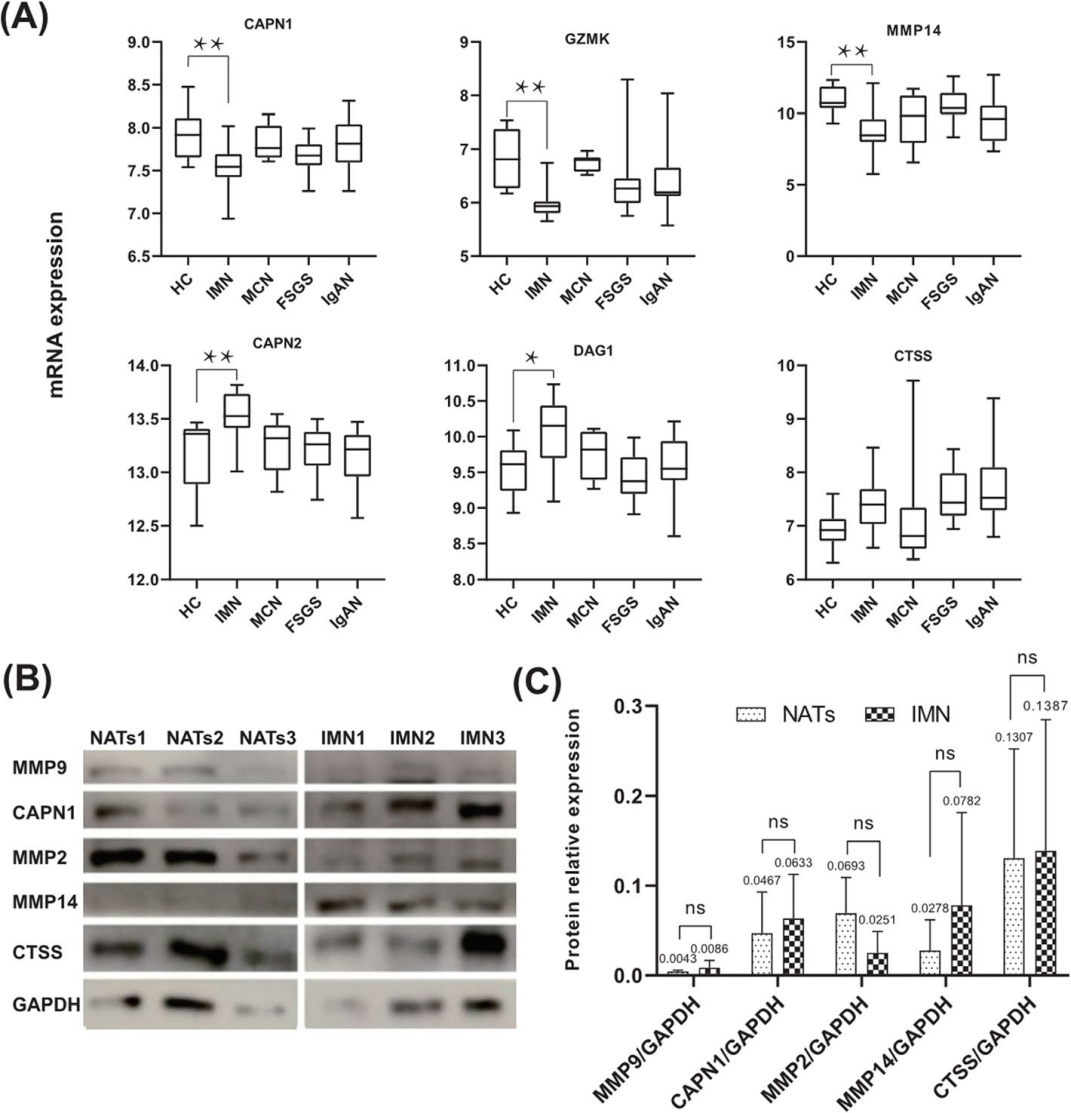
Protease validation using Gene Expression Omnibus (GEO) database and western blot analyses. **A** Proteases differentially expressed in idiopathic membranous nephropathy (IMN). **B** and (**C**) Western blot showing relative expression of proteases. ^*^ *P* < 0.05; ^**^ *P* < 0.01; ns: *P* > 0.05

Dysregulation of proteases in glomeruli may be the cause underlying the production of urinary peptides. Therefore, we explored the expression patterns of predicted proteases in the GSE99339 dataset based on GEO. This dataset contains mRNA expression data of 27 protease genes related to different kidney diseases. Compared with the HC group, the IMN group showed lower expression of *CAPN1*, *GZMK*, and *MMP14* (*P* < 0.05) and higher expression of *CAPN2* and *DAG1* (*P* < 0.05). Although *CTSS* expression was higher in the IMN group, the difference was not statistically significant (Fig. 6 A). This result suggested that the glomerular protease spectrum of IMN is different from that of HC, leading to pathological changes in glomerular function.

### Validation of proteases in IMN kidney tissue

Different proteases, such as calpain, cathepsin, and matrix metalloproteinases, are reportedly dysregulated in CKD [19–21]. The gelatinases, MMP2 and MMP9, mediate fibrosis in CKD, such as in diabetes mellitus [21]. Therefore, the expression of CAPN1, MMP2, MMP9, MMP14, and CTSS in kidney tissues of 3 IMN and 3 NATs were detected using western blotting (Fig. 6B). Compared with NATs, the IMN kidney tissue showed higher expression of CAPN1, MMP14, and CTSS; however, this difference was not statistically significant (*P* > 0.05) (Fig. 6 C). MMP2 and MMP9 levels also showed no apparent differences between the two groups (Fig. 6 C). These results suggest that specific proteases are dysregulated in kidney tissues of patients with IMN. Nevertheless, further validation of this protease dysregulation is warranted.

## Discussion

In this study, we examined urinary peptides and identified peptide-matched proteins to reveal the protein dysregulation related to IMN. To achieve high-throughput screening and save costs incurred by MS, we adopted the mixed sample strategy. Some studies have used capillary electrophoresis-MS or MALDI-TOF MS to analyze the peptides in human samples and identified the amino acid sequences. However, they also matched the identified peptide with corresponding proteins and studied the functions of the proteins [22, 23]. Here, we aimed to elucidate the production of urinary peptides and related proteases. Therefore, enriched urinary peptides were analyzed using routine proteomic methods, and matched proteins were identified easily.

Our results showed that 248 peptide-matched proteins were unique to IMN. It is generally believed that under the “label-free” quantitative strategy, there will be a 10–50 % loss of quantitative information of peptides and proteins, possibly because the content of some peptides is below the detection limit of the MS [24]. Therefore, it can be considered that the peptides were identified due to their high abundance. According to quantitative analysis, 31 proteins were dysregulated specifically in IMN. Thus, this study shows differences in urinary peptides among IMN patients, healthy individuals, and individuals with other kidney diseases, consistent with previous reports stating that the urine proteome can distinguish distinct subtypes of different disorders and disease conditions [25].

Studies have been conducted on urine proteomics of IMN [26, 27]. Ngai et al. [26] found that the function of 37 IMN urinary DEPs was involved in the signaling pathways related to the regulation of renal filtration. Pang Lu et al. [27] showed that the screened IMN urinary DEPs were associated with complement activation and coagulation cascade reactions. Besides the proteins mentioned above, the IMN urinary DEPs selected in this study were also involved in regulating proteasome, lysosome, actin cytoskeleton, antigen processing and presentation, and other signaling pathways, which may cause the production of endogenous peptides. However, the research on understanding the role of proteases and peptidomics in IMN is still very limited.

Proteases are necessary to maintain tissue homeostasis, and their dysregulation may be related to the production of urinary peptides in CKD [3, 9, 10]. Proteasix, a bioinformatics tool, is dedicated to exploring proteases that produce peptides. Brondani et al. [28] and Krochmal et al. [15] predicted various proteases related to the production of urinary peptides in diabetes using Proteasix. In this study, 32 proteases involved in IMN urinary peptide production were predicted by Proteasix. Further validation via western blotting indicated that the levels of proteases CAPN1, MMP14, and CTSS were higher in kidney tissue of IMN, whereas those of the proteases MMP2 and MMP9 did not change significantly. This was not completely consistent with the expression patterns of predicted protease in the GEO dataset, indicating that gene and protein expression trends might not be completely consistent [29]. This study proved that proteases uniquely dysregulated in IMN may be involved with urinary peptide production and that several proteases may be the driving factors of disease progression, which should be studied further.

There are mainly three intracellular proteolytic systems in the body: calpain, lysosome, and ubiquitin-proteasome system [30]. Calpains are a group of neutral cysteine proteases that are specifically activated by calcium ions. Calpain family members not only participate in protein hydrolysis and cytoskeleton remodeling but also cell cycle regulation and apoptosis [31, 32]. The balance of protein metabolism maintained by the calpain system will be disrupted by increased Ca^2+^ concentration. The abnormal or maladjusted activation of calpain is related to some pathological conditions [32].

Transient receptor potential cation channel 6 (TRPC6), a member of transient receptor potential cation channel protein superfamily [33], expressed in glomerular podocytes [40], is the most important ion channel mediating calcium influx in podocytes [33].

In addition, TRPC6, nephrin, and podocin constitute the podocyte SD complex, which maintains the normal function of the glomerulus, and its dysregulation causes proteinuria and glomerular diseases [33, 34]. Reiser et al. found that the expression level of TRPC6 mRNA in IMN patients was significantly higher than that in normal individuals [35]. Furthermore, the bound anti-PLA_2_R antibody and target antigen may activate Ca^2+^ influx and/or promote TRPC6 expression [35].

Therefore, elevated intracellular Ca^2+^ levels enhance calpain activity, which may destroy podocytes and other cells. Studies have shown that increased intracellular Ca^2+^ levels activate calpain, which can hydrolyze cytoskeletal proteins actin [36], cleave apoptosis-related proteins Bax, activate caspase 3, and then cause cell death or apoptosis [37]. Linkermann et al. [38] pointed out that calpain was involved in programmed cell necrosis in acute kidney injury. Yamashima et al. [39] put forward the hypothesis of “calpain-cathepsin.” Ischemia and hypoxia activate calpain that, in turn, acts on lysosomes, changing or disrupting lysosomal membrane permeability, leading to cathepsin in lysosomes being released into the cytoplasm. Recent studies have shown that the physical interaction between TRPC6 and podocyte calpain regulates podocyte cytoskeleton, cell adhesion, and movement [20].

Lysosomes are involved in cell processes such as secretion, cell signal transduction, and energy metabolism to maintain cell stability [40]. The kidney is the organ with the highest lysosome content; the integration of lysosome membrane protein and soluble lysosome hydrolase mediates lysosome activity. New evidence shows that lysosome protease cathepsin (CTSS) plays an essential role in the pathogenesis and progress of kidney diseases [19, 41]. The main physiological function of CTSS is beyond lysosomes, and it participates in the degradation of extracellular matrix proteins [19].

The western blot validation experiment in this study suggested that CTSS elevated in the kidney of IMN patients. At the same time, Jobs et al. [42] found that the high circulating level of CTSS was related to the increased risk of human death due to CTSS involving complex pathways leading to impaired renal function. In addition, the upregulation of CTSS was detected in ochratoxin A-induced nephropathy [43], indicating that the findings of this study agree with previous work, suggesting that the high CTSS expression in IMN may be of great significance. It has also been found that, in mice, serum CTSS level and markers of inflammation-related endothelial dysfunction, such as soluble tumor necrosis factor receptor (sTNFR1, sTNFR12), increase with the decrease of eGFR. By contrast, in humans, eGFR increase is related to a reduction of CTSS [44]. These findings indicate that CTSS activity increases with CKD progression, thus representing a potential marker of disease progression.

Matrix metalloproteinases (MMPs) can hydrolyze extracellular matrix (ECM) and cell adhesion molecules [21], which play a key role in renal fibrosis. Increasing evidence shows MMP imbalance in acute kidney injury, diabetic nephropathy, and glomerulonephritis [45].

The main roles of MMP-2 and MMP-9 are degrading collagen type IV [46], inducing the loss of cell connection, and promoting epithelial-mesenchymal transition (EMT) of tubular cells, which leads to tubular atrophy and fibrosis [45]. MMP14 light in activate MMP-2 and MMP-9 [47] and has non-proteolytic function. These molecules participate in all stages of the inflammatory process and renal fibrosis, eventually leading to the gradual decline of renal function [46, 48]. IMN is often accompanied by renal fibrosis, and we found that the expression of MMP-14 increased significantly in IMN, but no obvious changes were found in MMP-2 and MMP-9. Some studies confirmed that the activity and abundance of MMP-2 and MMP-9 decreased in the early stage of diabetic nephropathy; therefore, renal function damage may be related to the decrease in MMP-2 and MMP-9 activities.

The protease network is very complex, and some factors may hinder the relationship between natural peptides and kidney damage. The relationship between endogenous peptides and proteases should be carefully explained based on experiments. This study revealed that the dysregulation of urinary peptides in IMN might be related to proteases, providing clues for understanding the basic mechanism of IMN and exploring the therapeutic targets. However, this study still has some shortcomings: First, pooled samples were used in the study. Because the production of urine protein is affected by pre-kidney, the kidney itself, and post-kidney factors, interindividual differences will interfere with experimental results. Second, the amino acid sequence of a peptide has not been identified, which cannot explain the role of a certain peptide and its corresponding protease in diseases. Third, the mRNA and protein expression levels of proteases are not completely consistent. Therefore, the correlation analysis between the 24-h urine protein quantification, serum creatinine, cystatin C, and the expression level of proteases may explain the significance of proteases in kidney function injury. The lack of this analysis is also a deficiency, which shall be addressed in future studies.

## Conclusions

Our findings provide valuable insights to deepen our understanding of the role of proteases in IMN and highlight the potential significance of calpain, cathepsin, and MMPs in the pathogenesis of this disease.

## Methods

### Subjects

Random urine samples for peptidomics were collected from patients with biopsy-proven IMN (*n* = 22), minimal change disease (MCN, *n* = 5), focal segmental glomerulosclerosis (FSGS,* n* = 5), and IgA nephropathy (IgAN, *n* = 5), treated at the Shengjing Hospital of China Medical University from February 2019 to October 2019. Random urine samples from healthy controls (HC, *n* = 20) were simultaneously collected. All patients were aged 18–75 years; the sex and age of the HCs matched those of the case group; HCs met the criteria for normal blood routine, urine routine, and kidney and liver function tests. Patients with kidney disease secondary to other diseases, autoimmune diseases, endocrine system diseases, tumors, and pregnancy complicated with kidney diseases and those receiving immunosuppressant or hormone therapy were excluded. Urine samples were divided into three groups: HC, IMN, and other kidney disease (OD, including MCN, IgAN, and FSGS).

Kidney tissue samples of patients with biopsy-proven IMN (*n* = 3) and adjacent non-cancerous tissues (NATs, *n* = 3) from the biological sample bank of Shengjing Hospital of China Medical University were used for validation experiments. IMN samples were sourced from patients who met the above inclusion and exclusion criteria. This study was approved by the Ethics Committee of Shengjing Hospital of China Medical University (No. 2019PS194K); the requirement for informed consent of the patients was waived by this committee. All methods were carried out in accordance with relevant guidelines and regulations.

### Urine collection and urinary peptide extraction and digestion

Random urine samples were collected in the morning into 10 mL polypropylene centrifuge tubes, avoiding mixing with pollutants, and centrifuged at 1,500 rpm for 5 min at room temperature. The supernatant was stored at -80 ℃. Next, 200 µL of urine sample was concentrated, and 10 µL of pure water was added to it to dissolve the protein. Peptides were extracted using weak cation magnetic beads (WC-MB) according to the manufacturer’s instructions (Yixinbochuang, Beijing, China). Extracted peptides were stored at -80 ℃.

Urinary peptides extracted from HC, IMN, and OD groups were pooled into group-specific samples and then analyzed via a bottom-up proteomics strategy. The identified proteins were termed peptide-matched proteins. Dithiothreitol (Sigma, Oakville, Canada) was added to the sample to obtain a final concentration of 5 mM and then reduced at 56 ℃ for 30 min. Iodoacetamide (VETEC, Shanghai, China) was added to a final concentration of 11 mM and incubated for 15 min at room temperature in the dark. Trypsin (Promega V5111, Madison, WI) was added at a ratio of 1:50 (protease: protein, m/m) and incubated overnight at 37 ℃. Then trypsin was added at a ratio of 1:100 (protease: protein, m/m) and incubated for a further 4 h. The salts were then removed from peptides using Strata X (Phenomenex, USA), and the peptides were redissolved in TEAB/water (1:1 v/v) (SIGMA, Buchs, Switzerland).

### Matrix-assisted laser desorption/ionization time-of-flight MS (MALDI-TOF-MS)

Peptide fingerprint of each sample (not pooled) was profiled using MALDI-TOF-MS on a Clin-TOF-II instrument (Clin-TOF-II, Yixinbochuang, China). First, 1 µL of extracted peptide eluate was spotted onto the AnchorChip target and allowed to air dry. Next, 1 µL of matrix solution containing α-cyano-4-hydro-xycinnamic acid (Yixinbochuang, China) was spotted onto the same spot and air-dried and ionized using a nitrogen laser (λ = 337 nm) operating at 40 Hz. After running the MALDI-TOF-MS, mass calibration was performed. For each MALDI spot, 500 spectra were acquired (100 laser shots at five different spot positions) and processed using BioExplorer software.

### DIA-MS

Peptides were dissolved in mobile phase A for liquid chromatography and separated with a NanoElute ultra-high-performance liquid chromatography system (Germany, Bruker). Mobile phase A was an aqueous solution containing 0.1 % formic acid and 2 % acetonitrile. Mobile phase B was a solution containing 0.1 % formic acid and 100 % acetonitrile. Liquid-phase gradient settings were as follows: 0–70 min, 6–24 % B; 70–84 min, 24–35 % B; 84–87 min, 35–80 % B; 87–90 min, 80 % B; flow rate maintained at 450 nL/min. The peptide was separated using an ultra-high-performance liquid-phase system, injected into a capillary ion source for ionization, and then analyzed using MS (timsTOF Pro, Bruker, Germany). The ion source voltage was set at 2.0 kV, and the parent ions of peptide segments and their secondary fragments were detected and analyzed by high-resolution TOF. The scanning range of secondary MS was set to 100–1700. Parallel accumulation serial fragmentation (PASEF) mode was used in the data acquisition mode. A secondary spectrogram with the charge number of parent ions in the range of 0–5 was collected 10 times in the PASEF mode after collection of the primary mass spectrum, and the dynamic exclusion time of tandem MS scanning was set to 30 s to avoid repeated scanning of parent ions.

### Data analysis

Raw data were searched against Maxquant 1.6.6.0. The setting of retrieval parameters was as follows. The database was Homo_sapiens_9606(20,366 sequences), and an anti-database was added to calculate the false discovery rate (FDR) caused by random matching. The quantitative method was set to label-free quantification (LFQ), and the FDR of protein identification and peptide-spectrum match (PSM) identification was set to 1 %. The quantitative calculation method of protein was as follows. The signal abundance of proteins in each sample was detected using MS. The LFQ intensity of proteins in each sample was obtained using the LFQ calculation method. The ratio of protein LFQ intensity between two different samples was considered the differential expression ratio for the comparison group.

### Bioinformatics analysis

Gene Ontology (GO) and Kyoto Encyclopedia of Genes and Genomes (KEGG) signaling pathway enrichment analyses of proteins were carried out using the DAVID 6.8 online tool [49–52] (https://david.ncifcrf.gov/home.jsp). The interaction network of biological processes was analyzed and visualized using Cytoscape 3.8.1 [53], ClueGO 2.5.7 [54] software, Wukong cloud online platform [55], and GraphPad Prism 8.0 software.

### Protease prediction

Proteasix [56] is an online open-source protease prediction tool focused on peptides and is specially used to predict the cleavage site of proteins and the corresponding proteases. In brief, Proteasix uses the information about natural peptides, UniProt ID, and the starting and ending amino acid positions of corresponding proteins to predict potential proteases. Proteasix retrieves information about cleavage sites from protease databases (MEROPS, BRENDA) and considers restriction of cleavage sites (from ENZYME database). Most human proteases have several cleavage sites, i.e., one peptide sequence can be cut by different proteases.

### Validation based on database analysis

#### Analysis of tissue source of urinary peptides

The open-source sc-RNA seq database Single Cell Portal contains a single-cell sequencing dataset. SCP211 dataset [57] containing single-cell sequencing data of normal adult kidneys was selected to analyze the tissue source of urinary peptide-matched proteins related to IMN. The cell types involved in this dataset are podocytes, endothelial cells, pericytes, proximal convoluted tubular cells (PCTs), proximal straight tubular cells (PSTs), thin descending limb (TDL), thin ascending limb (TAL), collecting duct-principal cells (CD-PCs), collecting duct-A-intercalated cells, CD-A-ICs), distal convoluted tubule (DCT), mesangial cells (Mes), fibroblasts (Fib), venous smooth muscle cells (vSMC), macrophages, natural killer cells (NKs), and T cells. If the protein was not expressed in the above kidney cells, it was considered that the protein in the urine was not from the kidney.

#### Validation of differentially expressed proteins in urine and kidney tissues of patients with IMN

iProX [58] is an open-source integrated proteomics resource; the dataset IPX0002813000 was produced from our laboratory under the scope of another project whose results have not yet been published. This dataset contains data from kidney tissues of IMN patients and normal controls. We analyzed the expression levels of urinary peptide-matched proteins in the kidney tissues based on this dataset.

#### Validation of protease expression in the glomerulus

The preprocessed gene expression data set GSE 99,339 [59] was downloaded from the Gene Expression Omnibus (GEO) database (https://www.ncbi.nlm.nih.gov/GEO/). This dataset contains mRNA expression data of kidney glomeruli obtained using laser microdissection from patients with FSGS (*n* = 12), IgAN (*n* = 26), MCN (*n* = 8), and IMN (*n* = 21) and HCs (*n* = 8). The expression patterns of protease genes in different kidney diseases were examined.

#### Western blot analysis

To extract the proteins from the tissues, 3 IMN kidney tissues and 3 NATs were cut into pieces and lysed on ice using RIPA lysis buffer (Applygen, China) supplemented with Protease Inhibitor Cocktail, and then quantified using the BCA Protein Assay Kit. According to the expression of the internal reference protein GAPDH in the specimen, 90 µg extracted proteins from IMN kidney tissue and 10 µg extracted proteins NATs were separated by 12 % SDS-PAGE and transferred to a nitrocellulose (NC) membrane (Millipore, Darmstadt, Germany). After blocking with 5 % non-fat dry milk in tris-buffered saline and 0.1 % Tween 20 solution, membranes were incubated overnight at 4 ℃ with primary antibodies against the following proteins: MMP9 (ABGENT, China, 1:1000), CAPN1 (Abclonal, China, 1:1000), MMP9, MMP14, CTSS, and GAPDH (Proteintech, China, 1:5000). The membranes were then incubated with the appropriate HRP-Goat Anti-Rabbit (Elabscience, China, 1:2,500) and HRP-Sheep Anti-Mouse (Jackson ImmunoResearch, USA, 1:8,000) secondary antibody for 1 h at room temperature. The specific bands were detected using an ECL detection kit (Applygen, China) and captured on an ImageQuant LAS 4000 mini system (GE Healthcare, NJ, USA). Relative expression was determined by normalizing to GAPDH expression using the ImageJ software (Version 1.51j, National Institutes of Health, MD, USA).

### Statistical analysis

GraphPad Prism 8.0 was used for statistical analysis. Chi-square test was used to compare gender composition among groups; one-way analysis of variance (ANOVA) was used to compare differences between groups for quantitative indicators. Data are presented as mean ± standard deviation. *P* < 0.05 was considered statistically significant.

## Supplementary information

**Additional file 1**

**Additional file 2**

**Additional file 3**

**Additional file 4**

**Additional file 5**

## Data Availability

All data generated or analyzed in this study are included in this manuscript and its supplementary information files. Gene expression profiles GSE 99,339 was available from the Gene Expression Omnibus (GEO) database (https://www.ncbi.nlm.nih.gov/GEO/).

## Abbreviations

IMN: Idiopathic membranous nephropathy
DIA-MS: Data-independent acquisition mass spectrometry
GEO: Gene Expression Omnibus
PLA_2_R: Phospholipase A_2_ receptor
CKD: Chronic kidney disease
sc-RNA seq: Single-cell RNA sequencing
MCN: Minimal change disease
FSGS: Focal segmental glomerulosclerosis
IgAN: IgA nephropathy
NATs: Non-cancerous adjacent tissues
KDIGO: Kidney Disease Improving Global Outcomes
Cr: Creatinine
CysC: Cystatin C
eGFR: Estimated glomerular filtration rate
CAPN1: Calpain 1
CTSS: Cathepsin S
MMP: Matrix metalloproteinase
