# Supplementary material for: Urinary peptidomics reveals proteases involved in idiopathic membranous nephropathy
Source: BMC Genomics. 2021 Nov 24;22:852. doi: 10.1186/s12864-021-08155-3 (PMC8613922; doi:10.1186/s12864-021-08155-3)
Supplement: Supplementary file 4 — Additional file 4 [file 12864_2021_8155_MOESM4_ESM.docx]

For WB validation:

CAPN1, MMP2, MMP9, MMP14, GAPDH were detected in one NC membrane, then, CTSS was detected with the regenerated NC membrane.

(One NC membrane was cut into 2 pieces: 1.CAPN1, MMP2, MMP9, MMP14; 2.CTSS)

Image Multiple exposures of WB: MMP9, CAPN1, MMP2, MMP14. (**5 times**)

No 1.


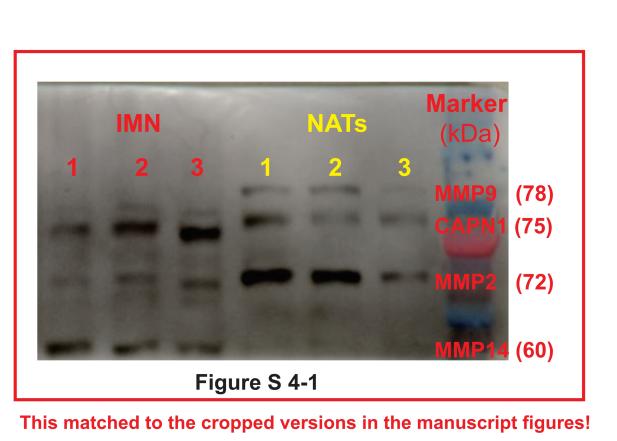


Figure S 4-1. The first time exposure of gel for MMP9, CAPN1, MMP2, MMP14.

No 2.


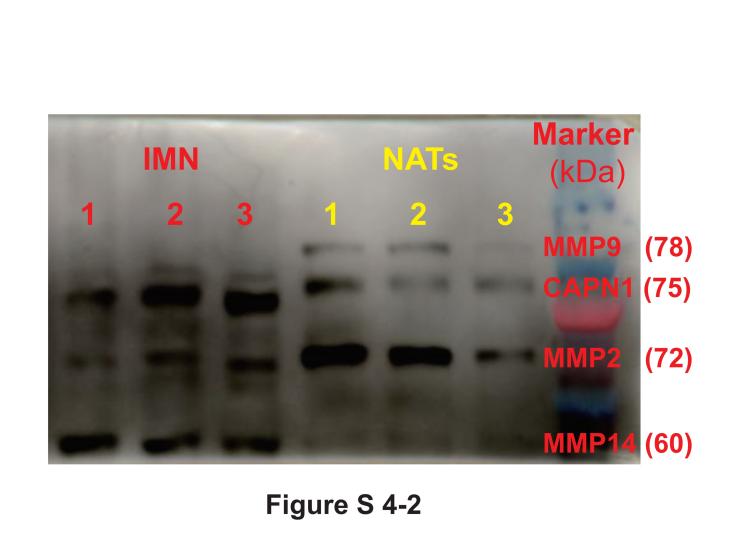


Figure S 4-2. The second time exposure of gel for MMP9, CAPN1, MMP2, MMP14.

No 3.


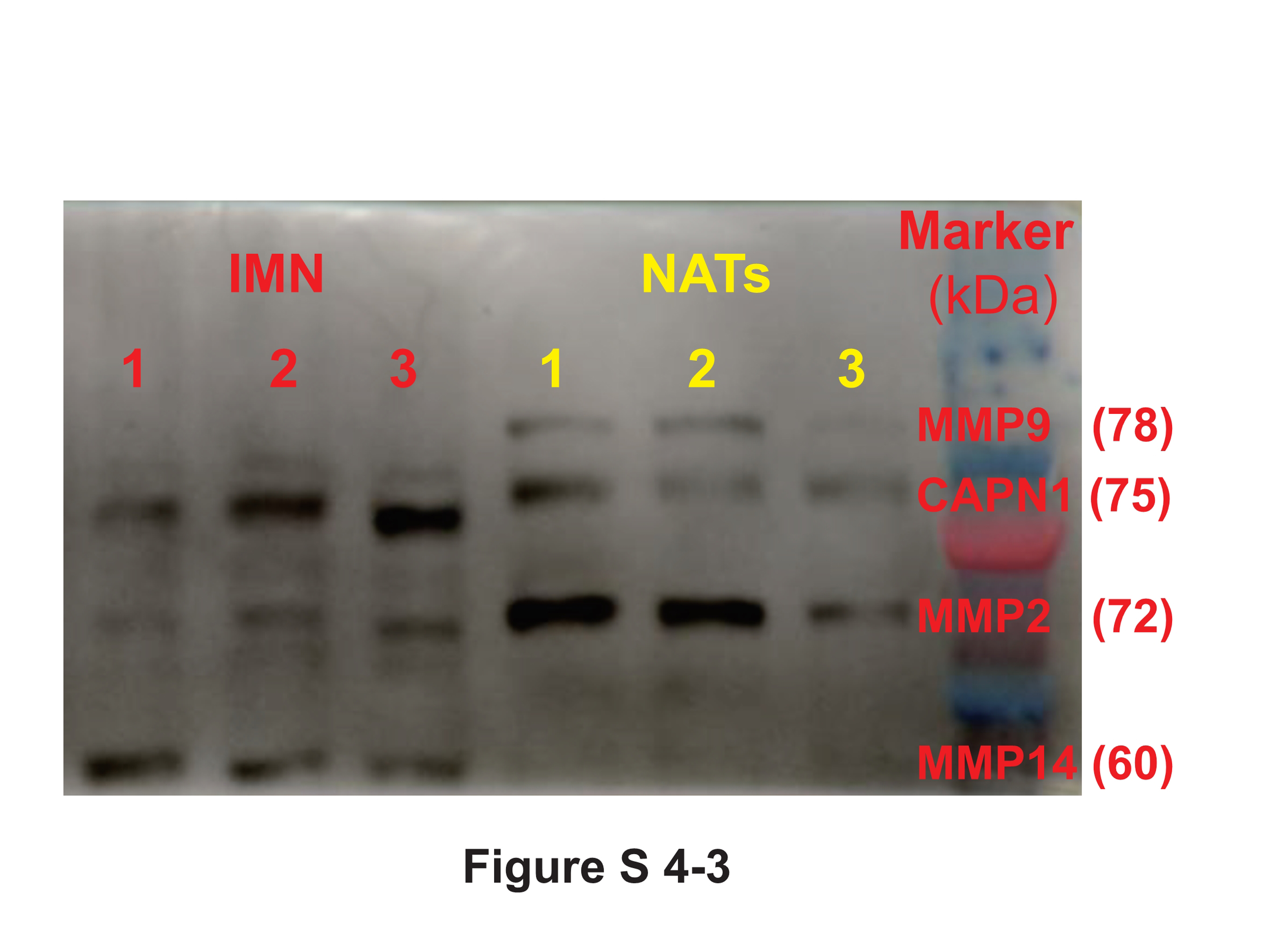


Figure S 4-3. The third time exposure of gel for MMP9, CAPN1, MMP2, MMP14.

No 4.


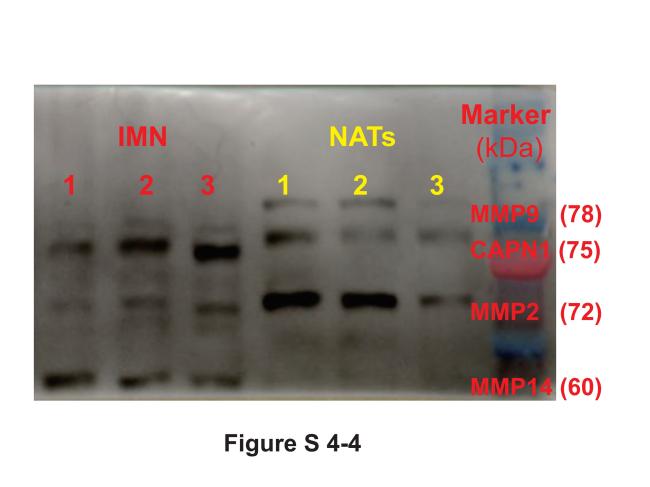


Figure S 4-4. The second time exposure of gel for MMP9, CAPN1, MMP2, MMP14.

No 5.


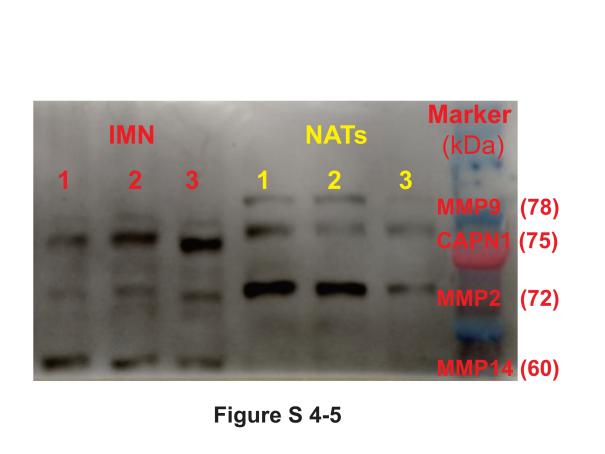


Figure S 4-5. The fifth time exposure of gel for MMP9, CAPN1, MMP2, MMP14.

Image Multiple exposures of WB: CTSS. (4 times)

No 1.


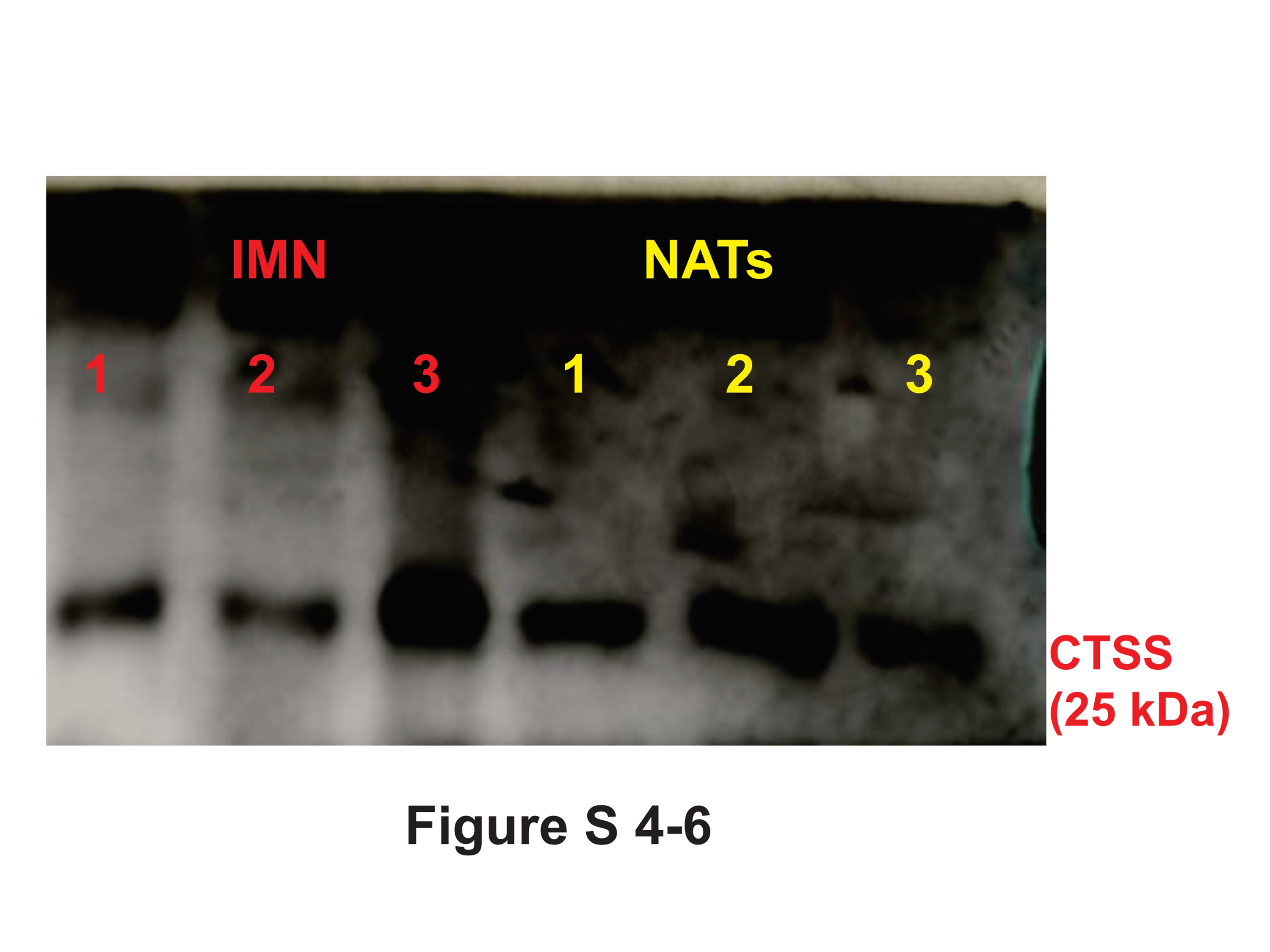


Figure S 4-6. The first time exposure of gel for CTSS.

No 2.


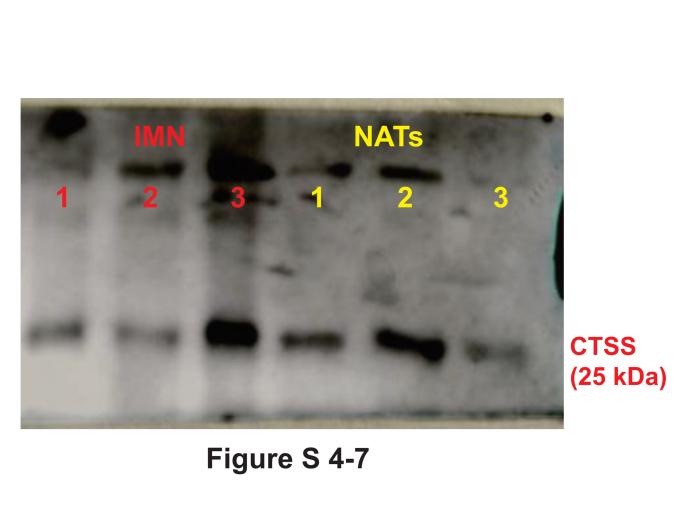


Figure S 4-7. The second time exposure of gel for CTSS.

No 3.


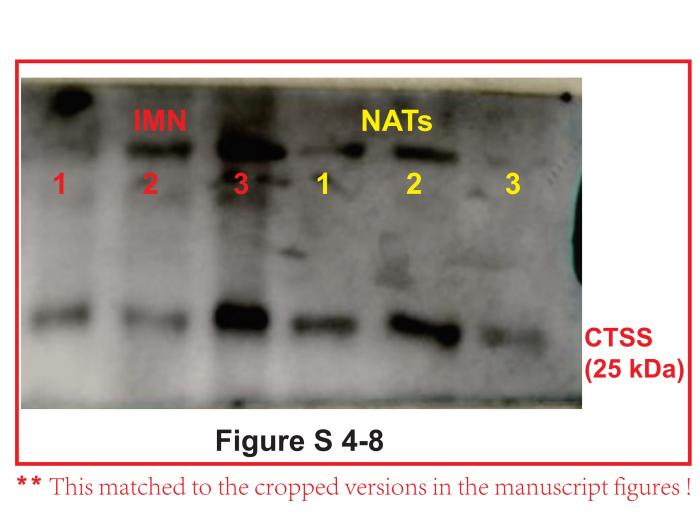


Figure S 4-8. The third time exposure of gel for CTSS.

No 4.


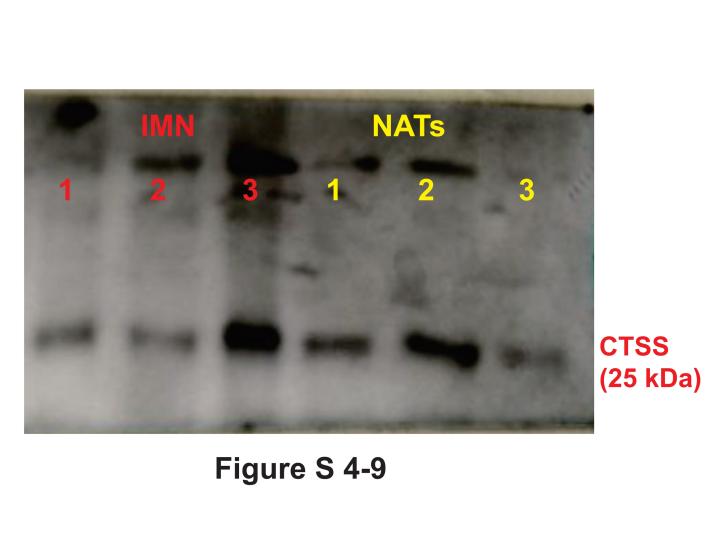


Figure S 4-9. The forth time exposure of gel for CTSS.

Image Multiple exposures of WB: GAPDH. (2 times)

No 1.


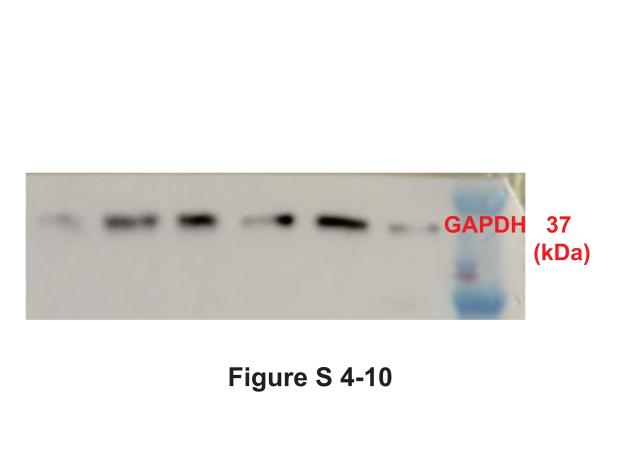


Figure S 4-10. The first time exposure of gel for GAPDH.

No 2.


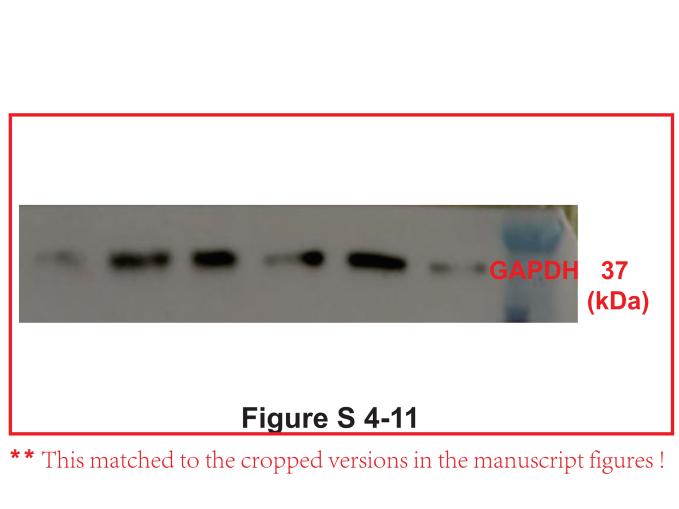


Figure S 4-11. The second time exposure of gel for GAPDH.
