# Supplementary figures and images for: Urinary peptidomics reveals proteases involved in idiopathic membranous nephropathy
Source: BMC Genomics. 2021 Nov 24;22:852. doi: 10.1186/s12864-021-08155-3 (PMC8613922; doi:10.1186/s12864-021-08155-3)

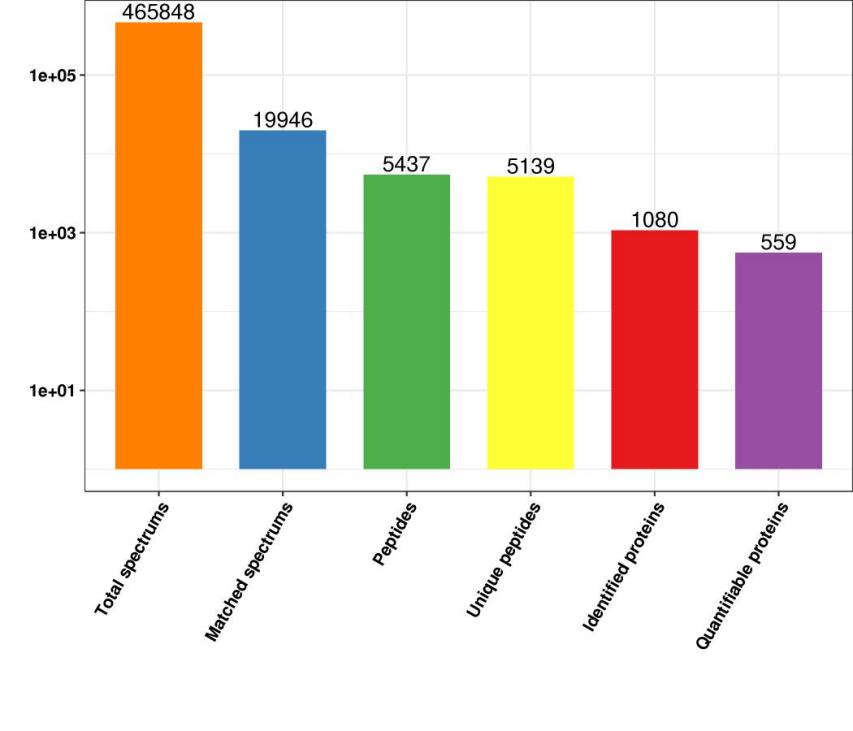


Figure S1.Overview of protein identified. 559 quantifiable proteins were identified by DIA-MS.

Supplement: Supplementary file 5 — Additional file 5 [file 12864_2021_8155_MOESM5_ESM.docx]
